# DPY-17 and MUA-3 Interact for Connective Tissue-Like Tissue Integrity in *Caenorhabditis elegans*: A Model for Marfan Syndrome

**DOI:** 10.1534/g3.115.018465

**Published:** 2015-04-27

**Authors:** Pauline Fotopoulos, Jeongho Kim, Moonjung Hyun, Waiss Qamari, Inhwan Lee, Young-Jai You

**Affiliations:** * Department of Biochemistry and Molecular Biology, Virginia Commonwealth University, Richmond, Virginia 23298; † Department of Biological Sciences, Inha University, Incheon, 402-751, South Korea

**Keywords:** Marfan syndrome, connective tissue, Fibrillin-1, molting, *mua-3*

## Abstract

*mua-3* is a *Caenorhabditis elegans* homolog of the mammalian *fibrillin1*, a monogenic cause of Marfan syndrome. We identified a new mutation of *mua-3* that carries an in-frame deletion of 131 amino acids in the extracellular domain, which allows the mutants to survive in a temperature-dependent manner; at the permissive temperature, the mutants grow normally without obvious phenotypes, but at the nonpermissive temperature, more than 90% die during the L4 molt due to internal organ detachment. Using the temperature-sensitive lethality, we performed unbiased genetic screens to isolate suppressors to find genetic interactors of MUA-3. From two independent screens, we isolated mutations in *dpy-17* as a suppressor. RNAi of *dpy-17* in *mua-3* rescued the lethality, confirming *dpy-17* is a suppressor. *dpy-17* encodes a collagen known to genetically interact with *dpy-31*, a BMP-1/Tolloid-like metalloprotease required for TGFβ activation in mammals. Human *fibrillin1* mutants fail to sequester TGFβ2 leading to excess TGFβ signaling, which in turn contributes to Marfan syndrome or Marfan-related syndrome. Consistent with that, RNAi of *dbl-1*, a TGFβ homolog, modestly rescued the lethality of *mua-3* mutants, suggesting a potentially conserved interaction between MUA-3 and a TGFβ pathway in *C. elegans*. Our work provides genetic evidence of the interaction between TGFβ and a fibrillin homolog, and thus provides a simple yet powerful genetic model to study TGFβ function in development of Marfan pathology.

Marfan syndrome is an autosomal dominant connective tissue disorder and is among the most frequent (3/10,000) monogenic diseases worldwide (Ramirez and Dietz 2007; Gao *et al.* 2010). Patients exhibit connective tissue and skeletal defects such as elongated extremities, joint hypermobility, scoliosis, striae, and chest wall deformities (Ramirez and Dietz 2007).

Marfan syndrome results from mutations in the *fbn1* gene (Dietz *et al.* 1991; Pyeritz 2000), which encodes an extracellular matrix (ECM) glycoprotein, Fibrillin-1 (FBN1) (Corson *et al.* 1993). FBN1 is a major component of the FBN1-rich microfibrils, a scaffold of elastic fibers (Kielty *et al.* 2005). Intact activity of FBN1 is critical to maintain the aortic wall structure, which consists of elastic fibers, collagen fibrils, and smooth muscle cells (SMCs). However, in Marfan patients, these structures are defective and altered due to reduced activity of FBN1 (Cui *et al.* 2014). As a result, Marfan syndrome patients die mostly due to aortic aneurysm, dissection, and rupture (Judge and Dietz 2005).

Among multiple functional domains, FBN1 contains nine TGFβ binding domains that are highly homologous to latent TGFβ binding proteins (Isogai *et al.* 2003; Hubmacher *et al.* 2006). Once secreted, FBN1 monomers aggregate and form beaded structures, which form macro-aggregates, microfibrils (Ramirez and Dietz 2007). Within these microfibrils, FBN1 sequesters TGFβ and BMPs in the ECM (Isogai *et al.* 2003; Sengle *et al.* 2008; Ramirez and Rifkin 2009). In patients with Marfan syndrome, defects in FBN1 lead to excess TGFβ signaling, which results in the pleiotropic manifestations of disease (Ramirez and Dietz 2007).

TGFβ is involved in cell proliferation, differentiation, apoptosis, migration, immunity, angiogenesis, ECM production, and the development of many organs (Massague 2000; Massague and Chen 2000; Massague and Wotton 2000). TGFβ activation occurs following the release from the ECM, which is mediated by metalloproteases such as BMP-1 (Ge and Greenspan 2006; ten Dijke and Arthur 2007). Active TGFβ binds to its serine/threonine transmembrane receptors, which in turn phosphorylate SMADs to regulate the transcription of genes (Neptune *et al.* 2003).

The *Caenorhabditis elegans* genome contains two clear homologs of human FBN1, *fbn-1* and *mua-3* (Culetto and Sattelle 2000; Bercher *et al.* 2001; Frand *et al.* 2005). MUA-3 localizes to hypodermal cells, to which body wall muscle adheres and which are required for adhesion of the hypodermis to the cuticle (Plenefisch *et al.* 2000**;** Bercher *et al.* 2001**).** A defect in *mua-3* is characterized by progressive muscle detachment throughout larval development (Bercher *et al.* 2001). *fbn-1* is required for proper molting, specifically during the L3/L4 molt and L4/young adult molt (Frand *et al.* 2005). Complete knockout of *mua-3* is lethal, whereas *fbn-1* knockdown mutants exhibit molting defects, sterility, larval lethality, and slow growth, and lay dead eggs (Bercher *et al.* 2001; Frand *et al.* 2005). Mutations in *fbn-1* and *mua-3* genes cause molting defects that can be attributed to defects in a tissue that is functionally equivalent to connective tissue in mammals (Bercher *et al.* 2001; Frand *et al.* 2005). This demonstrates that *C. elegans* fibrillin genes have a conserved role in maintaining connective tissue-like tissue integrity, as does human FBN1. The molting defects of these *C. elegans* mutants suggest that the mechanical strain of molting (shedding and rebuilding the exoskeleton) mimics Marfan syndrome pathology where weak connective tissue is torn.

Interestingly, in *C. elegans* most genes that regulate body size are components of a TGFβ pathway (Patterson and Padgett 2000). Because alteration in body size is a prominent indicator of Marfan syndrome, it suggests that connective tissue-like tissue integrity mediated by *mua-3* or *fbn-1* could be related to TGFβ signaling pathways in *C. elegans* because they are implicated in Marfan syndrome.

In this study, we identified a new allele of *mua-3* that causes death specifically during the L4 molt in a temperature-dependent manner. Using this phenotype, we performed unbiased genetic screens and found DPY-17 as a genetic interactor of MUA-3. We also found a potential interaction between MUA-3 and a TGFβ ligand DBL-1, suggesting a potential conservation in interaction between a fibrillin homolog and a TGFβ pathway. Our study could be used to further establish a worm model of Marfan syndrome.

## Materials and Methods

### *C. elegans* strains and culture conditions

*C. elegans* were routinely grown on NGMSR plates (Avery 1993). All animals were maintained at 20° on *E. coli* HB101 (Sulston and Hodgkin 1988) except for *mua-3(uy19)* (YJ35) mutants, which were maintained at 15°. The wild-type strain was *C. elegans* variant Bristol, strain N2. Mutant strains used are YJ35 *mua-3(uy19) III*, OT136 *mua-3(rh195) III*, CB164 *dpy-17(e164) III*, CB1072 *unc-29(e1072) I*, DP38 *unc-119(ed3) III*, CB1282 *dpy-20(e1282ts) IV*, CB369 *unc-51(e369) V*, RB1547 *sta-2(ok1860) V*, NU3 *dbl-1(nk3) V*, and YJ208 *mua-3(uy19) III*; *dbl-1(nk3) V*.

### Mapping of the new *mua-3* mutation

#### SNP mapping:

The death phenotype during the L4 molt was called drop-dead, Drd. To identify the chromosome on which the *drd* mutation resides, the mutant was crossed to four mutants: *unc-29 I*, *unc-119 III*, *dpy-20 IV*, and *unc-51 V*. F2s homozygous for the marker from each cross produced approximately 20% Drd except the cross to *unc-119* (<3%). This mapped *drd* to chromosome III. Mating *unc-119(ed3)* with Drd homozygotes yielded wild-type F1 progeny, indicating the mutation is recessive. Thirteen out of 14 F2 Unc produced no Drd progeny (F3), validating that *drd* is linked to *unc-119* on chromosome III. One Unc produced progeny with both Unc and Drd phenotypes by recombination. This *unc-119(ed3) drd (uy19*) grows normally at 15°. We used it for further SNP mapping. The *unc-119(ed3) drd (uy19*) double mutants mated with Hawaiian CB4856 males produced wild-type progeny (F1). We placed 48 F2 Unc singly onto plates, and then the DNA of F3 progeny was collected for further SNP mapping (Wicks *et al.* 2001; Davis *et al.* 2005). Because *unc-119* is located at 10.9 Mb, SNP markers on ZK1236 (8.4 Mb), C07A9 (9.6 Mb), T21C12 (10.5 Mb), Y39A1A (10.6 Mb), and Y75B8A (12.0 Mb) were selected. One hundred percent (44/44) of Unc progeny carried only the N2 marker (0% Hawaiian) at 12.0 Mb, suggesting that the mutation is located to the left of *unc-119*. Forty-eight percent (21/44) of Unc progeny carried only the N2 marker at T21C12 (10.5 Mb) and 58% (26/45) of Unc progeny carried only the N2 marker at Y39A1A (10.6 Mb). Based on the recombination frequencies, we estimated the *drd* location at approximately 10.1–10.2 Mb.

#### Whole genome sequencing:

The genomes of the mutant strains after outcrossing twice against N2 were sequenced using Illumina sequencing. Sequences were aligned to the WS220 reference *C. elegans* genome with bowtie2 (Langmead and Salzberg 2012), variants called with the samtools/bcftools suite (Li *et al.* 2009), and effects on gene function predicted and variants filtered with snpEff and SnpSift (Cingolani *et al.* 2012). Further specific analyses used vcftools (Danecek *et al.* 2011), bedtools (Quinlan and Hall 2010), and custom scripts.

After whole genome sequencing, we found a single deletion in the *mua-3* gene, but no other mutations in the range of 9.8 Mb to 10.5 Mb for chromosome III. The mutation was also validated because the original strain was from the knockout consortium of *C. elegans*, where the mutations are generated by the UV/TMP method to create deletions. The new allele of *mua-3(uy19)* contains a 488-bp in-frame deletion that includes parts of the 30^th^ and 31^st^ exons and the short intron between them. The flanking sequences are as follows: 5′ end of the deletion: 5′-TGAGGAGAATGGATA-3′ and 3′ end of the deletion: 5′-CACCACAGTCCAGTC-3′. The deletion results in deletion of the following 131 amino acids:

RCRCRNGYHD DDPAHPGHRC SFMINECDSS NLNDCDRNAN CIDTAGGYDC ACKAPYRDEG PPQSPGRICR LNECLNPNRN TCDRNADCRD LDYGYTCTCR HGFYDQSPNP QEPGRICIEF QQEEHIERVK V.

### Characterization of *mua-3* phenotypes

#### Temperature-dependent lethality:

Eggs were isolated via hypochlorite treatment (Sulston and Hodgkin 1988) and then harvested in M9 buffer to obtain a synchronous population. Synchronized populations of YJ35 *mua-3(uy19)* mutants and wild-type were transferred to *E. coli* HB101 seeded NGM plates at the L1 stage and cultivated at 15°, 20°, or 25° in triplicate. Synchronized populations of approximately 100 *mua-3* and wild-type L1s were placed onto each plate. At 25°, approximately 30 hr after L1s were given food, L4s started to molt. At 20°, L4s started to molt after approximately 40 hr, and at 15° L4s started to molt after approximately 60 hr. The majority of death occurred during the molt; therefore, timeframes for the experiments were designated accordingly. The number of dead animals and the number of total animals were counted to determine percent survival of *mua-3* and wild-type at each hour from 50 hr at 15°, 37 hr at 20°, and 30 hr at 25°.

#### Sterility:

To determine the number of offspring produced by individual animals of OT136 *mua-3(rh195)* mutants and wild-type, five L4s of each strain were transferred singly to *E. coli*-seeded NGM plates and their progeny were counted. Each worm was transferred to a new plate every day to avoid crowding and to visualize all the progeny easily. Progeny were counted 3 d later.

### Suppressor screens

#### Primary screen:

*mua-3(uy19)* were collected at the L4 stage and were randomly mutagenized with ethyl methanesulfonate (EMS) (Brenner 1974). P_o_ animals were plated onto *E. coli* HB101 seeded plates and moved to 15°. F1 progeny were synchronized and remained at 15°. The homozygous F2 generation was synchronized and moved to 25°. The surviving animals were isolated.

#### Secondary screen:

Individual suppressors of the *mua-3* mutant lethality were isolated and moved to individual plates at the restrictive temperature, 25°. Sterile animals and escapers (animals that produced more than 90% of nonviable progeny) were removed. A total of 20 F2s passed the secondary screen and were used to found lines maintained at 25°.

#### Complementation test for Dpy suppressors:

Unidentified suppressor hermaphrodites were crossed with wild-type males. The F1 progeny males were crossed with an alternate unidentified suppressor. The F1 progeny of this cross were counted to calculate the percentage of Dpy. The percentage of males in the population was also counted to determine whether Dpy progeny were from cross-fertilization or self-fertilization. If 50% of the progeny were Dpy, then suppressors failed to complement each other.

### RNAi of *dbl-1* and *dpy-17*

The bacteria-mediated feeding RNAi was performed as described (Fraser *et al.* 2000), with the following modifications. The *mua-3*(*uy19*) mutants were fed with the clones of *dbl-1* and *dpy-17* from the Ahringer feeding library (Fraser *et al.* 2000; Kamath and Ahringer 2003). The plates containing NGM agar with 1 mM IPTG and 50 mg/ml carbenicillin were inoculated with bacterial cultures grown 16–18 hr for each targeted gene. L4-stage *C. elegans* were transferred to the plates for each gene at 15°. Forty-eight hours later, adults were removed and the plates were placed at 25°. Forty-eight hours later, the number of progeny that survived was counted and the percentage was calculated. The RNAi clones of *dpy-17* and *dbl-1* were sequenced and validated (data not shown).

### Photography

*mua-3* mutants were observed under differential interference contrast (DIC) microscopy using a Zeiss Axio A2 Imager at either 63× or 100× magnifications. Images were acquired using Zeiss Axiovision software.

### Statistical analysis

Student's *t* tests and one-way ANOVA test were performed to determine statistical significance.

### *mua-3* homology comparison

The phylogenetic tree was created using the MEGA program. CLUSTALW was used for multiple sequence alignment. The *p-distance* method was used to calculate distance and the neighbor-joining method was used to create a tree. The tree was statistically evaluated by using the bootstrap method (1000 times), and robustness of the tree branch was indicated at the branch. The following protein sequences were used for the phylogenetic analysis: *C. elegans* MUA-3 (NP_001022674.1), *C. elegans* MUP-4 (NP_498645.1), *C. elegans* FBN-1 (NP_001263711.1), *Drosophila melanogaster* DP (Dumpy; NP001260037.1), *Homo sapiens* FBN1 (NP_000129.3), FBN2 (NP_001990.2), FBN3 (NP_115823.3), and EYS (Eyes shut; NP_001278938.1). *H. sapiens* EYS sequence was included as an outgroup member to infer the root of this unrooted tree.

## Results

### Characterization of a *mua-3(uy19)* mutation

**Figure 1:**
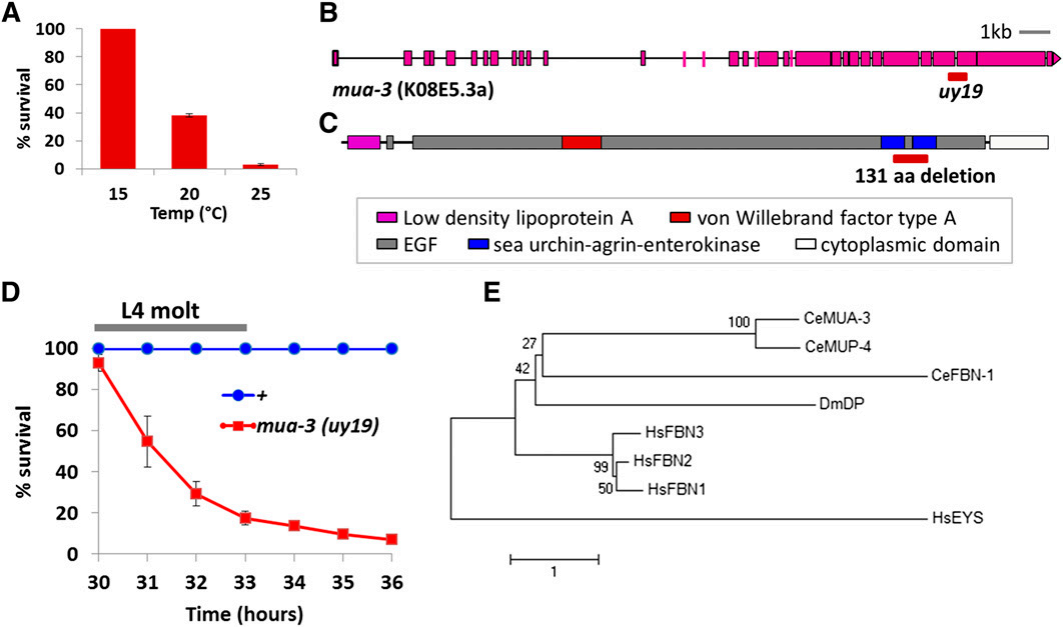
Characterization of a newly isolated *mua-3* allele, *uy19*. (A) Temperature-dependent lethality of the newly isolated *mua-3* mutant. The numbers of dead animals and total animals were counted to calculate percent survival. Animals were synchronized and plated as L1s and the percentage of survival was measured after they passed the L4 molt; 61 hr for 15°, 40 hr for 20°, and 30 hr for 25°. The numbers are average percent survival ± SEM. (B) A schematic drawing of the exons and introns of *mua-3* gene and the location of the deletion. (C) A schematic drawing of the domain structure of the *mua-3* gene (Bercher *et al.* 2001) and the location of the deletion. (D) Time course of death of the *mua-3(uy19)* mutants at 25°. Very few deaths were observed until 30 hr from L1. Then, within 3–4 hr, more than 90% were dead. The time window of death was somewhat wider than a worm’s molting period (approximately 2 hr) because the mutants are not perfectly synchronized, and thus the time to reach the molt varies among the animals. The numbers are average percent survival ± SEM. (E) A phylogenetic tree to show MUA-3 is a homolog of human fibrillins. Compared fibrillin homologs are *C. elegans* MUA-3, *C. elegans* MUP-4, *C. elegans* FBN-1, *Drosophila melanogaster* DP, *Homo sapiens* FBN1, FBN2, and FBN3. *H. sapiens* EYS sequence was included as an outgroup member to infer the root of this unrooted tree.

We acquired RB1547 from the Caenorhabditis Genetics Center (CGC), which contains a deletion in *sta-2*. We observed a high rate of lethality during the L4 molt at 25° (Figure 1A). After outcrossing against N2 two times, we found the lethality was not linked to the deletion in *sta-2*. After a series of SNP mappings, we located the lethal mutation on chromosome III close to *unc-119*. Subsequent whole genome sequencing revealed a 488-bp (131 amino acids) in-frame deletion in the *muscle attachment abnormal*-3, *mua-3* gene (see *Materials and Methods*) (Figure 1, B and C).

The mutants showed a lethal phenotype similar to the previously reported *mua-3* mutants, confirming that the lethality is due to a mutation in *mua-3* (Bercher *et al.* 2001). From transmission electron microscopy (TEM) observations of detachment zones of their *mua-3* mutants, Bercher and others suggested that MUA-3 acts at the apical hypodermal surface to maintain the attachment of the hypodermis to the basal cuticle and that the primary defect in *mua-3* mutants is the failure of these attachments. But unlike the old allele *mua-3(ar62)* that die during various stages as early as at L1 (Bucher and Greenwald 1991), the new mutants grow normally until the L4 molt without any visible defects in development, and within 2–3 hr of molting, most die (Figure 1D) (30 hr to 33 hr from L1s is when most of the death occurs). At the L1, L2, and L3 stages, mutants rarely died. The occasional escapers after the L4 molt looked pale and sick immediately after molting, but within 2 hr they appeared normal and reproduced normally (data not shown).

**Figure 2:**
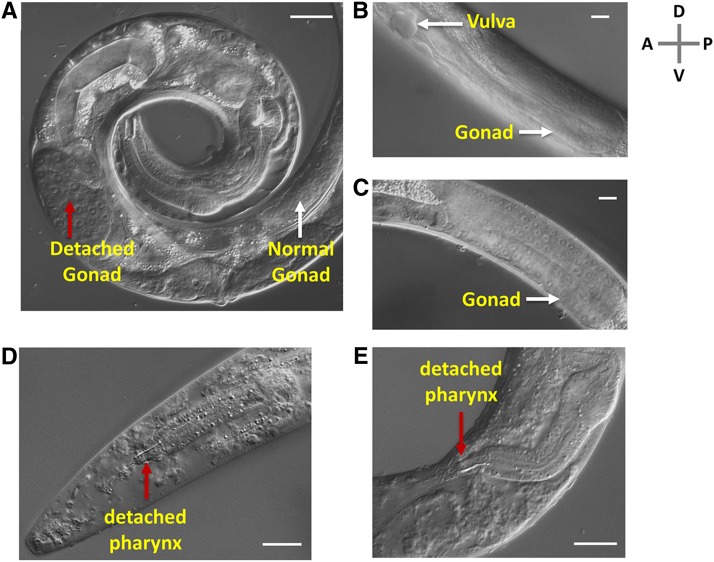
The phenotypes of *mua-3*. (A) Representative DIC microscopy image of *mua-3(uy19)* mutants dying during L4 molt. White arrow shows a normal-looking gonad arm with germ cell nuclei. The red arrow shows misplaced and detached gonad. Scale bar = 30 µm. A, anterior; P, posterior; D, dorsal; and V, ventral. (B and C) The gonad structures of *mua-3(uy19)* at early L4 stage before molt show normal development of gonads at 25°. Scale bar = 10 µm. (D and E) The tips of the pharynxes of two dying *mua-3(uy19)* were detached from the tips of the mouths. Scale bar = 20 µm

When we examined the cause of sudden death during L4 molt, we found that internal organs such as the pharynx and gonad were detached and misplaced (Figure 2, A, D, and E). The gonad develops normally before molting at nonpermissive temperatures (Figure 2, B and C). This shows that MUA-3 is required for the organ attachment, consistent with the previous finding by Bercher *et al.* 2001. The sudden death during the L4 molt could suggest that mechanical strain during the L4 molt is sufficiently stressful to cause all the tearing in our mutants.

The sequence of MUA-3 shares high homology with that of the human FBN1, mutations in which are the cause of Marfan syndrome (Figure 1E). Our results may suggest that the detachment of internal organs during the L4 molt could mimic certain aspects of Marfan pathology such as aneurysm or aortic dissection in the heart caused by weak connective tissue integrity under mechanical stress.

### *dbl-1*, a homolog of TGFβ2, genetically interacts with *mua-3*

**Figure 3:**
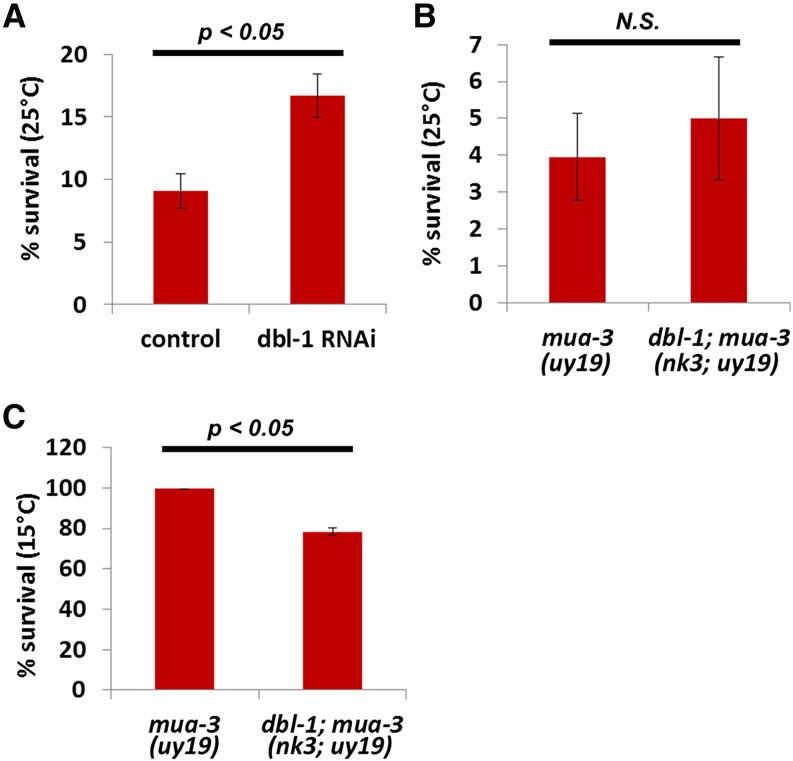
*dbl-1* genetically interacts with *mua-3* mutants. (A) RNAi of TGFβ2 homolog (*dbl-1*) rescued the *mua-3* lethality at 25°. The numbers are average percent survival ± SEM. (B) A mutation in *dbl-1* did not rescue the *mua-3* lethality at 25°. (C) A mutation in *dbl-1* reduces the survival of *mua-3* at 15°.

Because the sequence of *mua-3* is most similar to the human *fibrillin1* gene, and because the phenotype of our new mutant mimics certain aspects of Marfan pathology, we investigated the potential genetic interaction between *mua-3* and TGFβ2 signaling. Recent studies show that overactivation of the TGFβ2 pathway results in Marfan pathology and appearances such as connective tissue defects and long stature (for review see Lindsay and Dietz 2011). Interestingly, overexpressing DBL-1, a homolog of TGFβ2, increases body size in *C. elegans* (Morita *et al.* 2002). We hypothesized that the DBL-1 TGFβ pathway regulates body size in *C. elegans* as TGFβ2 does in humans, and thus in the *mua-3* mutants increased TGFβ signaling may contribute to lethality. To test this hypothesis, we knocked down the expression of *dbl-1* by RNAi and examined whether *mua-3* lethality was reduced. RNAi reduced the lethality of *mua-3* mildly yet significantly, suggesting a potential conserved link between excess TGFβ signaling and misregulation in body size shown in Marfan syndrome in *C. elegans* (Figure 3A). We generated double mutants of *mua-3*; *dbl-1* to further examine the interaction between two genes. The existing allele of *dbl-1(nk3)* carries a deletion covering the entire gene, and thus no protein is made. The double mutants did not survive any better than *mua-3* single mutants at 25° (Figure 3B). Interestingly, however, the double mutants die more at 15°, clearly showing a genetic interaction between two genes (Figure 3C). That the null mutation of *dbl-1* cannot rescue *mua-3* but RNAi knockdown of *dbl-1* can suggest that complete knockout of *dbl-1* does not help *mua-3*, but reduction of MUA-3 levels does. Also, the increase of the death in the double mutants shows there is genetic interaction between these two. This is fully consistent with the findings in mammals. Reduction of TGFβ2 by various means alleviates Marfan syndrome. However, conditional yet complete knockout TGFβ2 receptor in smooth muscle cells impairs the contractile apparatus of vascular smooth muscle and exacerbates the aortic disease induced by mutation of fibrillin1 (Li *et al.* 2014).

### Two independent alleles of *mua-3* show temperature-dependent phenotypes

**Figure 4:**
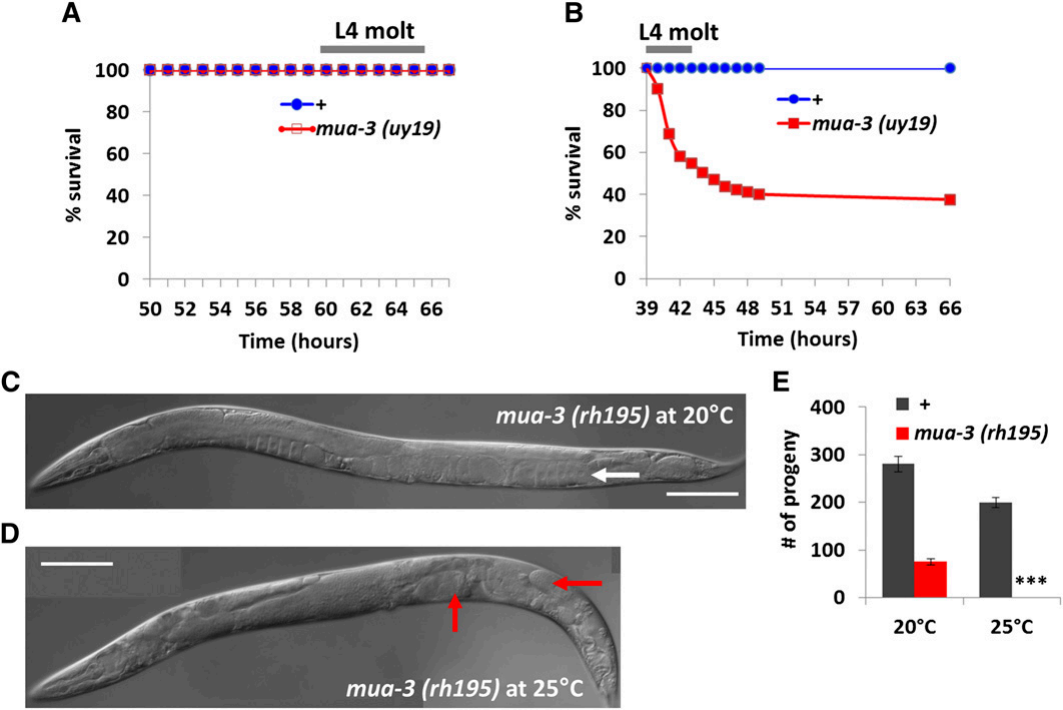
*mua-3* mutants showed temperature-dependent phenotypes. (A) *mua-3(uy19)* mutants do not show lethality at 15°. (B) Increase of temperature to 20° reduces survival of *mua-3(uy19)* mutants. (C and D) Another allele of *mua-3* mutants shows temperature-dependent sterility. *mua-3(rh195)* mutants show a normal gonad morphology at 20° (A, white arrow) but become sterile due to detachment of the gonad at 25° (D, red arrows). (E) The sterility induced by high temperature was quantified by counting the progeny of *mua-3* mutants and wild-type animals grown at 20° and 25°. ***P < 0.001 for comparison between the numbers of progeny of *mua-3* at 20° and 25° by Student's *t* test.

The lethality of *mua-3(uy19)* mutants was reduced when the mutants were grown at two lower temperatures, 15° and 20° (Figure 4, A and B). To examine whether the temperature sensitivity is specific to this mutation or could be seen in other *mua-3* mutations, we tested *mua-3(rh195)* mutants, the only viable *mua-3* mutant isolated by Bercher *et al.* (2001). Wild-type animals have fewer progeny at 25° than 20°. However, they do not show any sterility phenotype. On the contrary, *mua-3(rh195)* grew normally at 20° with reduced brood size but became completely sterile when grown at 25° due to gonadal detachment (see Figure 4, C–E for quantification of their sterility). This is a milder phenotype than that of *mua-3(uy19)*, which dies at the L4 molt, yet both mutations show severe phenotypes at a high temperature.

### Increased temperature manifests the reduction of body length in the *dbl-1* null mutant

**Figure 5:**
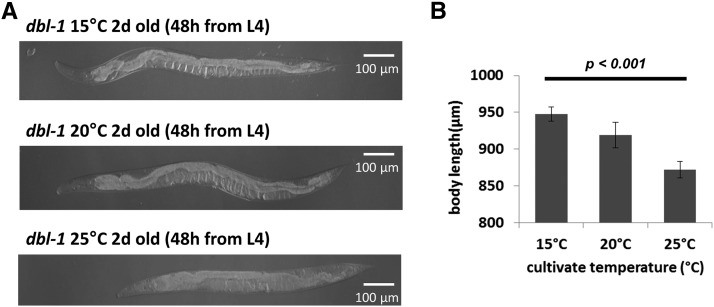
*dbl-1* null mutants showed temperature-dependent phenotypes. (A) The 2-day-old *dbl-1(nu3)* mutants grown at different temperatures show different body lengths; they are longest at 15° and shortest at 25°. (B) Quantification of the difference in body lengths. More than 20 mutants were used for each sample and the experiments were repeated three times. *P <* 0.001 by one-way ANOVA.

We noticed that the phenotype of *mua-3* is dependent on temperature, and so is that of the null mutant of *dbl-1*, the *C. elegans* TGFβ2 homolog. At 25° the mutants are shortest, at 15° the mutants are longest, and at 20° the body length is in between the two temperatures (25° and 15°) (see Figure 5, A and B for quantification). This allele does not produce any protein, yet the body length is more reduced at a high temperature. This result suggests that temperature influences the body size phenotype regardless of the stability or function of the protein. Moreover, similar to the case of *mua-3*, increased temperature exacerbates the phenotype.

### Mutations in *dpy-17* suppress *mua-3(uy19)* lethality

To identify genes that interact with *mua-3(uy19)*, we performed two independent unbiased genetic screens using ethyl methyl sulfonate (EMS) mutagenesis to identify suppressors. We used the temperature-dependent lethality of *mua-3(uy19)* to sort the resulting mutants of the genetic screens.

**Figure 6:**
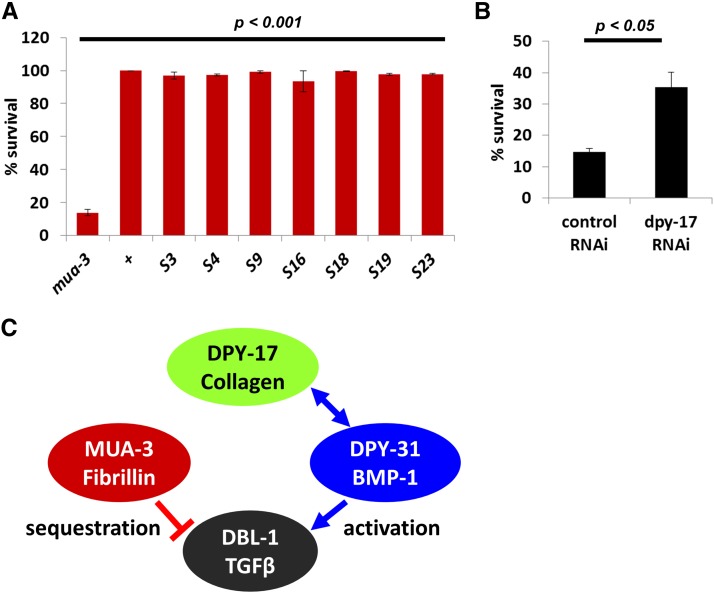
Mutations in *dpy-17* rescue *mua-3(uy19)* lethality. (A) Several suppressors were shown to suppress the *mua-3* lethality. The *P* values were calculated by comparing the percent survival of each suppressor with that of the *mua-3* mutants using the Student *t* test. (B) RNAi of *dpy-17* rescued the *mua-3* lethality, validating *dpy-17* suppression of *mua-3*. (C) A model to suggest potential interactions among *mua-3*, *dpy-17*, and *dpy-31* to regulate TGFβ signaling. Fibrillin-1, a human homolog of *mua-3*, sequesters TGFβ (DBL-1) to reduce the signal, whereas BMP-1/Tolloid metalloprotease (DPY-31) increases the signal. Based on the known interaction between DPY-17 and DPY-31 and its implication based on a function of DPY-31 homolog in mammals, it may be possible that the excess TGFβ (DBL-1) signal in the absence of MUA-3 could be ameliorated by reducing DPY-31 signal when DPY-17 was missing.

Among 20 isolates, seven suppressors were picked for further study after we removed weak suppressors, sterile mutants, and escapers (animals that produced nonviable progeny at nonpermissive temperatures) (Table 1). We confirmed their rescue by measuring survival at 25° (Figure 6A). Because the screens were performed with F2 progeny from mixed F1s, it is possible that some suppressors share the same mother and contain mutations on the same alleles; yet, several suppressors have distinct phenotypes. For example, S4, one of the strong suppressors (Figure 6A), does not show a Dpy phenotype but shows Eat (pale body color) and Unc (uncoordinated movement). Interestingly, several suppressors of *mua-3(uy19)* from two independent screens are smaller (Sma) or shorter (Dpy) in body length compared to wild-type. Because one of the major pathways that determines body size in *C. elegans* is a TGFβ pathway, our results suggest the intriguing possibility that MUA-3 may be involved in TGFβ regulation in *C. elegans* as in Marfan syndrome pathology in humans.

**Table 1:** Characterization of appearance of isolated suppressors of the *mua-3(uy19)* lethality

Suppressors	Phenotypes
S1	Small body size, pale body color, uncoordinated movement (Unc)
**S3**	Pale body color, small body size (Sma)
**S4**	Pale body color, uncoordinated movement (Unc)
S5	Uncoordinated movement (Unc)
S6	Small body size, pale body color
S7	Small body size, pale body color, uncoordinated movement (Unc)
**S9**	Short body length (Dpy), similar to *dpy-17* mutants
S10	Small body size
S11	Short body length (Dpy), similar to *dpy-17* mutants
**S16**	Slow growth, slow movement, small body size (Sma), pale body color
**S18**	Short body length (Dpy), similar to *dpy-17* mutants
**S19**	Short body length (Dpy), similar to *dpy-17* mutants
S20	Thin body width
S21	Long body length (Lon)
S22	Slow growth, slow movement, small body size (Sma), pale body color
**S23**	Short body length (Dpy), similar to *dpy-17* mutants
S24	Small body size (Sma)
S25	No obvious phenotype
S26	Dark body color
S27	Dark body color

S1–S11 are suppressors isolated from the first screen. S16–S27 are suppressors isolated from the second screen. The suppressors tested in Figure 3A are in bold.

Four suppressors (S11, S18, S19, S23) looked very similar to each other and shorter in body length than wild-type, exhibiting the Dpy phenotype. They failed to complement *dpy-17(e164)*, indicating that they all carry mutations in *dpy-17*. We isolated *dpy-17* mutations from two independently performed screens (S11 is from the first screen; S18, S19, and S23 are from the second screen), strongly suggesting that *dpy-17* mutations suppress *mua-3* lethality. S11 is close to sterile, probably due to other mutations; we excluded it from further study. To confirm *dpy-17* suppression of *mua-3* lethality, we treated *mua-3* mutants with *dpy-17* RNAi. *dpy-17* RNAi rescued *mua-3* lethality (Figure 6B), validating *dpy-17* as a suppressor. The maximum rescue rate we saw was approximately 50% survival. Only approximately 50% of wild-type animals treated with *dpy-17* RNAi showed a clear Dpy phenotype (data not shown), suggesting the low rescue percentage could be due to incomplete RNAi effect.

*dpy-17* encodes a cuticle collagen required for normal body morphology (Novelli *et al.* 2006). Genetic studies show that a gain-of-function mutation of *dpy-17* suppresses phenotypes of *dpy-31* mutations, indicating a genetic interaction between the two. *dpy-31* encodes a homolog of the human bone morphogenic protein-1 (Novelli *et al.* 2004). The human bone morphogenic protein-1 is a tolloid-like gene whose proteolytic activity is required for TGFβ activation (Ge and Greenspan 2006). Our screen results suggest a potential link between MUA-3-associated and TGFβ-associated molecules such as DPY-31 via DPY-17 (Figure 6C).

## Discussion

In this study, we isolated a new *mua-3* mutation that shows defects in connective tissue-like tissues that lead to death specifically during the L4 molt in a temperature-sensitive manner. The sequence homology and the similar phenotypes we and others found in *mua-3* mutants suggest that MUA-3 is a homolog of FBN1 and that the role of fibrillin genes in *C. elegans* and humans could be conserved. *mua-3(uy19)* lethality that is specific during the L4 molt (but not in other molts) could suggest that the mechanical strain may be most stressful during the L4 molt.

The temperature dependence shown in two independent alleles of *mua-3* is interesting. Despite the wide use of temperature-sensitive (*ts*) mutations, especially for studying essential genes that cause lethality when they are deleted, the molecular basis of *ts* phenotypes is largely unknown. It is generally assumed that the lethality results from thermal inactivation of gene products. However, there are also several lines of evidence suggesting that the exacerbated phenotypes at the nonpermissive temperature come from the changes in conditions under which the molecules function that coincide with the temperature shift (Van Voorhies and Ward 1999; Masuda *et al.* 2013), such as rates of metabolism or development processes. Our result that a null mutation of *dbl-1* has different body size depending on temperature also supports this idea. Therefore, higher temperature may exacerbate *mua-3* phenotypes by increasing metabolic rate and/or by speeding the developmental processes. One of the current treatments for Marfan syndrome is β-adrenergic blockers that lower heart rate and slow aortic growth. However, this only ameliorates the deleterious symptoms of progressive aortic root enlargement and aortic dissection. If metabolic rate plays any role in regulating disease progression, then a combined treatment with β-adrenergic blockers and a drug that decreases metabolic rate may help further to alleviate Marfan syndrome.

We identified *dpy-17* mutations as suppressors of *mua-3* from two independent genetic screens, suggesting that DPY-17 and MUA-3 genetically interact. DPY-17 encodes a cuticle collagen. *C. elegans* genome contains approximately 154 collagen genes, many of which are essential and developmental stage-specific (Johnstone 1994, 2000; Kramer 1997). In Marfan syndrome models, the tunica media of the aorta, which normally contains elastin sheets and collagen, is fragmented, disorganized, and lost (Cui *et al.* 2014). Also, it has been reported that collagen metabolism is abnormal in Marfan patients and overexpression of a collagen gene results in reduction of flexibility of extracellular matrix (ECM), thus contributing to Marfan pathology (Priest *et al.* 1973; Tajima 1995; Robinson *et al.* 2006). This might suggest that excessive collagen could negatively affect the structure of ECM when there is not enough Fibrillin-1, and that removal of DPY-17 when *mua-3* function is reduced might prevent the formation of abnormal ECM and rescue the lethality during the L4 molt.

DPY-17 is known to interact with DPY-31 (Novelli *et al.* 2006). DPY-31 is a homolog of bone morphogenic protein-1 metalloprotease. The human bone morphogenic protein-1 is a tolloid-like gene whose proteolytic activity is required for TGFβ activation (Ge and Greenspan 2006). In humans, increased activity of other metalloproteases such as MMP2 (metalloprotease 2) and MMP9 has clear implications in Marfan pathology; in Marfan syndrome, these endopeptidases cleave ECM components, including microfibrils, elastic fibers, and collagen, resulting in loss of ECM integrity and aggravation of the syndrome (Chung *et al.* 2007, 2008). Our study suggests that DPY-31 in *C. elegans* and BMP-1 in humans may be involved in TGFβ dysregulation, a process that has been implicated in Marfan syndrome disease progression (Ge and Greenspan 2006).

Together, our results suggest that Fibrillin-1, TGFβ, and a Tolloid-like protein may act in concert to modulate TGFβ availability and connective tissue-like tissue integrity in *C. elegans*. These results further suggest the molecular conservation among known genetic causes for Marfan or Marfan-related syndromes between humans and *C. elegans*, providing a useful genetic model for Marfan syndrome.
